# Supplementary material for: Anomalous Tunnel Magnetoresistance and Spin Transfer Torque in Magnetic Tunnel Junctions with Embedded Nanoparticles
Source: Sci Rep. 2015 Dec 18;5:18026. doi: 10.1038/srep18026 (PMC4683539; doi:10.1038/srep18026)
Supplement: Supplementary Information [file srep18026-s1.pdf]

SUPPLEMENTARY INFORMATION. *Scientific Reports* **5**, 18026; doi:10.1038/srep18026 (2015).

Anomalous tunnel magnetoresistance and spin transfer torque in magnetic tunnel junctions with embedded nanoparticles: supplementary information

Arthur Useinov[1,2,3], Lin-Xiu Ye[2], Niazbeck Useinov[3], Te-Ho Wu[4], and Chih-Huang Lai[2]

General model of the magnetic point contact

One can imagine metallic point-contact removing some pieces of the barriers $L_{1,2} \to 0$, Fig. 1 or (Fig. S1**a**). The metallic nanoparticle (NP) becomes a part of the top (left) and bottom (right) metallic FM layers connecting them together (transmission $D_s \to 1$). The charge current of the metallic point-contact with orifice cross section can be calculated according Eq. S1, which was derived in assumption that electron energy is equal to E_F, while $E_F >> k_B T$ [1]:

$$I_s = \frac{e^2 p_{F,s,\min}^2 a^2 V}{2\pi\hbar^3} \int_0^\infty dk \frac{J_1^2(ka)}{k} F_s(k, D_s, l_s), \tag{S1}$$

$$F_s(k, D_s, l_s) = F_s^{bal}(D_s) + F_s^{df'}(k, D_s, l_s) + F_s^{df''}(k, D_s, l_s), \tag{S2}$$

where e and l_s are electron charge and mean free path, respectively; a is radius of the spot-like contact and k_B is Boltzmann constant. k is Fourier image of radial variable ρ, which is the coordinate of electron on the contact plane; $J_1(x)$ is Bessel function. $p_{F,s,\min} = \hbar k_{F,s,\min}$ is value of the Fermi momentum, where $k_{F,s,\min}$ is Fermi wavenumber which has to be minimal (min) value between $k_{F,s}^L$ and $k_{F,s}^R$; $F_s(k, D_s, l_s)$ contains integration over $\theta_{c,s}$ which is angle between z-axis and direction of the electron velocity, where index $c = L(R)$ determines the contact side. D_s is transmission coefficient determined in range of standard definition of the quantum mechanics. Transmission usually is a function of the electron wavenumbers, $\theta_{c,s}$, applied voltage V and other possible variables. These variables have been defined from specification of the considered problems where point-contact area can be exchanged by quantum object, similar to the present problem.

All integrals inside Eq. S2 are written in relation to variable $\theta_{L,s}$ in order to simplify solution and integral limits. The total current is summation of the both spin components of the charge current $I = I_\uparrow + I_\downarrow$. The complete view of the first and second terms of Eq. S2 accessed in ref. 2 The first term has a simple form $F_s^{bal} = \left\langle \cos(\theta_{L,s}) D_s \right\rangle_{\theta_L}$ and is responsible for the ballistic and quantum-ballistic transports while $F_s^{df'}(k, D_s, l_s)$ and $F_s^{df''}(k, D_s, l_s)$ are responsible for quasi-ballistic ($l_\uparrow > a, l_\downarrow < a$ and $l_\uparrow, l_\downarrow \approx a$) and diffusive regimes of

[1]Department of Physics, National Tsing Hua University, Hsinchu, Taiwan. [2]Department of Materials Science and Engineering, National Tsing Hua University, Hsinchu, Taiwan. [3]Institute of Physics, Kazan Federal University, Kazan, Russian Federation. [4]Graduate School of Materials Science, National Yunlin University of Science and Technology, Douliou, Taiwan. Correspondence and requests for materials should be addressed to C.-H.L. (email: chlai@mx.nthu.edu.tw)

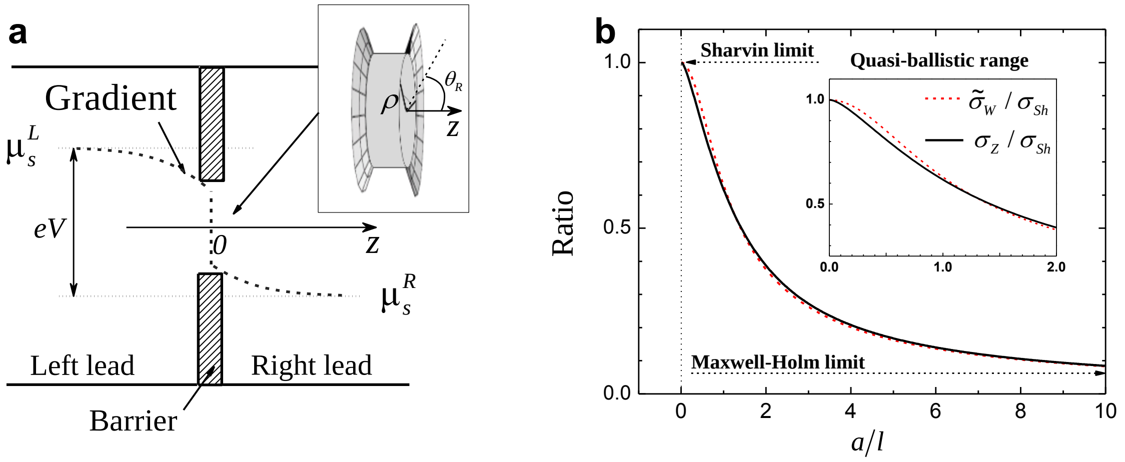

Figure S1. (**a**) Schematic plot of the point-like contact which is considered as an orifice in impenetrable wall (a -spot model); $\mu_s^{L(R)}$ is chemical potential. (**b**) shows numerical solution of the present model for σ_Z / σ_{Sh} and refined Wexler's ratio $\tilde{\sigma}_W / \sigma_{Sh}$ as function of a / l .

the conduction (or Ohmic resistance, $l_\uparrow, l_\downarrow << a$). All three terms of Eq. S2 are the solutions of the system for the quasi-classical Green functions with quantum boundary conditions. The last term $F_s^{df''}\left(k, D_s, l_s\right)$ considers the gradient of the chemical potential nearby contact interface (for more technical details check ref. 1).

It is worth to note that F_s^{bal} is not a function of k and l, and this fact is an important fundamental quantum property of the electron in nanoscale. If take only $F_s\left(k, D_s, l_s\right) = F_s^{bal}\left(D_s\right)$ then Eq. S1 can be simplified since $\int_0^\infty J_1^2\left(x\right)/ x dx = 1/2$, and:

$$\sigma_s = I_s / V = \frac{e^2}{h}\frac{k_{F,s,\min}^2 a^2}{2}\left\langle \cos\left(\theta_{L,s}\right) D_s \right\rangle_{\theta_L} . \tag{S3}$$

Equation S3 as a ballistic part of the general Eq. S1 shows validity of the Eq. 1. In case of symmetric *non*-magnetic point-like contact $k_F^L = k_F^R$, $\sigma_\uparrow = \sigma_\downarrow$, $D_\uparrow = D_\downarrow \to 1$, $F_s^{bal} = \left\langle \cos\left(\theta_{L,s}\right)\right\rangle_{\theta_L} = 1/2$, and finally: $\sigma_Z = \sigma_\uparrow + \sigma_\downarrow =$ $= (2e^2 / h)(k_F^2 a^2 / 4)$ which coincides with Sharvin conduction limit[3,4] σ_{Sh} . Thus, Eq. S3 is some kind of extension of Sharvin conduction limit and characterizes the quantum conduction of the quantum system which is located inside the contact area. The quantum physics of the system can be accounted through analytical or numerical view of the transmission. The conductance (Eq. S3) is different from "classical ballistic" one for the general case where $D_s \neq 1$, the definitions in terms of "coherent", "direct" or "quantum-ballistic" limits are more appropriated. Furthermore, in order to analyze the solution in range of the complete expression (Eq. S1) in the limit of the *non*-magnetic symmetric point-contact, all terms in Eq. S2 were simplified:

$$\sigma_Z = 4\sigma_{Sh}\left(\frac{1}{4} - \int_0^\infty \frac{dx}{x}\frac{J_1^2(x)}{1+\left(xK\right)^2 + \sqrt{1+\left(xK\right)^2}}\right) \tag{S4}$$

where $K = l / a$. In the limit when $K \to \infty$ ($a / l \to 0$), the integral is vanishing in Eq. S4 and conductance transformed into Sharvin limit.

Taking into account the exact integral's asymptotic for $K \to 0$ ($a/l \to \infty$):

$$\lim_{K\to 0}\int_0^{\infty}\frac{dx}{x}\frac{J_1^2(x)}{1+(xK)^2+\sqrt{1+(xK)^2}}=\frac{1}{4}-\frac{2}{3\pi}K$$

it is easy to obtain an exact diffusive solution (or Maxwell-Holm limit σ_M), $\sigma_Z \to \sigma_M = (8K/3\pi)\sigma_{Sh} = 2a/\rho_V$, where $\rho_V = \left(e^2 nl/\hbar k_F\right)^{-1} = \left(e^2 p_F^2 l/3\pi^2\hbar^3\right)^{-1}$ is resistivity in volume $[\Omega\cdot\mathrm{m}]$ and $n = k_F^3/3\pi^2$ is electron density in metals. The dependence of the numerical ratio σ_Z/σ_{Sh} on a/l is very close to Wexler solution $\tilde{\sigma}_W/\sigma_{Sh} = \left(\frac{3\pi}{8K}\gamma(K)+1\right)^{-1}$ with Mikrajuddin's corrections[5,6], where $\gamma(K) \approx \frac{2}{\pi}\int_0^{\infty} e^{-K\cdot x}\,\mathrm{sinc}(x)dx$. Figure S1**b** clearly reveals that our model shows good matching between Sharvin and Maxwell-Holm classical limits.

TEMPERATURE DEPENDENCE

Considering the *T*-impact, it is noted that the ballistic conductance shows lack of dependence on *T*. As a result, the thermal heat occurs relatively far away from the contact area, i.e. on the distance larger than mean free path. However, Ohmic resistance (diffusive regime) depends on *T* since metal resistivity is sensitive to the temperature[4]. Yet the temperature impact for the simulating NP in range of double barrier system might be considered as indirect *T*-dependence of the transmission, especially for $k_n \approx 0.1 - 0.46\,\text{Å}^{-1}$. Transmission D_s is a function of k_n and if the Fermi energy of NP is comparable with $k_B T$ then k_n has the margin of the values, which significantly changes the conductance behavior due to conduction band broadening.

The key parameter of our model is k_n. The consideration of the system in terms of finite temperature depends on how corresponding energy of k_n is compared to $k_B T$. In range of $k_n < 0.46\,\text{Å}^{-1}$ (corresponding energy $E_n = \hbar^2 k_n^2/2m$) the thermal energy at room temperature is important, while for $0.1 < k_n < 0.26\,\text{Å}^{-1}$ even a few Kelvins is important; therefore, we have to add additional integration over k_n in the following form to get more correct conductance:

$$G_s \propto \int_{X_1}^{X_2} dx \int_0^{\theta_{\min}} \sin(\theta_s)\cos(\theta_s) D_s\left(\theta_s, k_{n,s}+x\right) d\theta_s\,, \tag{S5}$$

where $X_{1(2)} = \mp\sqrt{2mk_B T/\hbar^2}$. With increasing temperature, the *T*-induced band broadening destroy TMR dips and peaks[7]. For example Fig. 3c, Fig. 3e and Fig. 3f in ref. 8 clearly show how the width of resonant peak increases with temperature at low voltages and its amplitude slowly decreases. However the resonant TMR peak has to be more stable for higher temperatures in contrast to TMR suppression since k_n is larger (e.g. Fig. 3f in ref. 8 reveals this fact). Our simulations show classical dome-like TMR behaviors without anomalies when $k_n > 0.5\,\text{Å}^{-1}$ and developed approach is valid at room temperature at this case.

ACKNOWLEDGMENT

The work was supported by MOST, Taiwan (grants No.104-2221-E-007-046-MY2, 103-2112-M-007-011-MY3) and RFBR (grant No. 14-02-00348-a). A. Useinov acknowledges partial support by the Program of Competitive Growth of Kazan Federal University.

References

1. Useinov, N. Kh. Semiclassical Green's functions of magnetic point contacts. *Theor. Math. Phys.-Engl. Tr.* **183,** 705-714 (2015).

2. Useinov, A., Deminov, R., Tagirov, L. & Pan, G. Giant magnetoresistance in nanoscale ferromagnetic heterocontacts. *J. Phys. Condens. Matter* **19,** 196215 (2007).

3. Sharvin, Y. V. A possible method for studying Fermi surfaces. *Sov. Phys.* **21,** 655-656 (1965).

4. Timsit, R. S. Electrical conduction through small contact spots. *Electrical Contacts.* (Proceedings of the 50th IEEE Holm Conference on Electrical Contacts and the 22nd International Conference on Electrical Contacts). 184 -191 (2004).

5. Wexler, G. The size effect and the non-local Boltzmann transport equation in orifice and disk geometry. *Proc. Phys. Soc.* **89,** 927-941 (1966).

6. Mikrajuddin, A., Shi, F., Kim, H. & Okuyama, K. Size-dependent electrical constriction resistance for contacts of arbitrary size: from Sharvin to Holm limits. *Mater. Sci. Semicond. Process.* **2,** 321-327 (1999).

7. A.N. Useinov, and C.H. Lai, Tunnel magnetoresistance and temperature related effects in magnetic tunnel junctions with embedded nanoparticles. *SPIN* (accepted Nov. 2015).

8. Ciudad, D. *et al.* Competition between cotunneling, Kondo effect, and direct tunneling in discontinuous high-anisotropy magnetic tunnel junctions. *Phys. Rev. B* **85,** 214408 (2012).
